# Archaea in the Human Microbiome and Potential Effects on Human Infectious Disease

**DOI:** 10.3201/eid3008.240181

**Published:** 2024-08

**Authors:** Stefanie Duller, Christine Moissl-Eichinger

**Affiliations:** Medical University of Graz, Graz, Austria (S. Duller, C. Moissl-Eichinger); BioTechMed Graz, Graz (C. Moissl-Eichinger)

**Keywords:** archaea, microbiome, human microbiome, pathogenicity, oral cavity, gastrointestinal tract, *Methanobrevibacter*, urogenital tract

## Abstract

Archaea represent a separate domain of life, next to bacteria and eukarya. As components of the human microbiome, archaea have been associated with various diseases, including periodontitis, endodontic infections, small intestinal bacterial overgrowth, and urogenital tract infections. Archaea are generally considered nonpathogenic; the reasons are speculative because of limited knowledge and gene annotation challenges. Nevertheless, archaeal syntrophic principles that shape global microbial networks aid both archaea and potentially pathogenic bacteria. Evaluating archaea interactions remains challenging, requiring clinical studies on inflammatory potential and the effects of archaeal metabolism. Establishing a culture collection is crucial for investigating archaea functions within the human microbiome, which could improve health outcomes in infectious diseases. We summarize potential reasons for archaeal nonpathogenicity, assess the association with infectious diseases in humans, and discuss the necessary experimental steps to enable mechanistic studies involving archaea.

Archaea are a group of single-celled microorganisms with distinct characteristics, and constitute 1 of the 3 domains of life, along with bacteria and eukarya. Although they were thought to live only in extreme environments such as hot springs, archaea have emerged as important components of the human microbiome. Despite their potential importance to human health and disease, archaea are studied less than other members of the microbiome, such as bacteria and fungi. The human microbiome harbors a variety of archaeal species from different phyla (*1*). Archaeal representatives have been found in various body sites, including the skin, the respiratory tract, the urogenital tract, and the gastrointestinal tract (*2*,*3*). Human-associated archaeal communities exhibit spatial patterns similar to those of bacteria; notable examples are the predominant signatures of ammonia-oxidizing Nitrososphaeria on the skin, methane-producing (methanogenic) Archaea in the urogenital and gastrointestinal tracts, and unknown Nanoarchaeota (formerly Woesearchaeota) in the respiratory tract (*1*–*3*).

Given that the gastrointestinal tract contains 99% of all microbial biomass in the human body and serves as the gateway for numerous diseases, research on human-associated archaea, as with bacteria, has primarily focused on fecal samples. In human feces, the most prevalent (>90%) archaeal representatives are *Methanobrevibacter smithii* and *Candidatus* Methanobrevibacter intestini, (*1*,*4*,*5*). In particular, *M. smithii* is highly abundant, especially in persons emitting high levels of methane (*6*). Because many studies primarily concentrate on fecal samples rather than in vivo samples, and because of general methodological challenges associated with archaea detection (*7*,*8*), our understanding of the human archaeome (all archaea residing in the human body) still lacks comprehensive insights into diversity, taxonomic classification, and, importantly, the functional and mechanistic roles of archaea.

## Pathogenic Traits 

Pathogenicity, the ability to cause disease in multicellular hosts, is widespread in various domains of life. In total, >1,500 human pathogenic bacterial species belonging to 10 phyla and 24 classes are known (*9*). The pure ratio observed in the bacterial domain indicates that >16 archaeal pathogens should exist (*10*); however, despite individual case reports and possible correlations with certain polymicrobial diseases (e.g., periodontitis), archaea are mostly considered nonpathogenic. No single representative of archaea known to date possesses capabilities consistent with Koch’s postulates, which has led to several discussions about possible reasons for that observation (*10*–*13*).

In theory, archaea have the prerequisites to evolve into pathogens because they are genetically and metabolically diverse, ubiquitous in the environment, and able to conflict with close relatives by using compounds directed against archaea (*10*). It is possible that our limited ability to accurately recognize them in disease conditions (not-yet-been-discovered hypothesis) is the reason no archaeal pathogens have been identified (*8*,*11*). Other perspectives have proposed that archaeal pathogens might not exist, potentially because they use different cofactors than those found in eukaryotes (*14*) or are unable to use the organic resources of the host, which are typically exploited by bacterial pathogens on a large scale (metabolism hypothesis) (*15*; D.J. Harrison, master’s thesis, 2022, https://researchspace.auckland.ac.nz/handle/2292/63414). Alternatively, some argue that pathogenesis in archaea does not exist because they do not possess the required virulence factor genes (gene-absence hypothesis) (D.J. Harrison, master’s thesis, 2022). Related hypotheses suggest that archaea cannot acquire virulence factors from bacteria or eukaryotes, possibly because their viruses and cell walls do not permit transfer between domains (virus hypothesis) (*10*). All those hypotheses warrant discussion. However, considering the huge lack of knowledge regarding gene annotation (virulence factors might be present, but we cannot identify them; or the metabolic capacities of methanogenic archaea have not yet been fully assessed, and we do not understand their behavior in the host with respect to metabolites taken up), a more superficial view on the principles of bacterial pathogenic traits might be useful.

**Figure 1:**
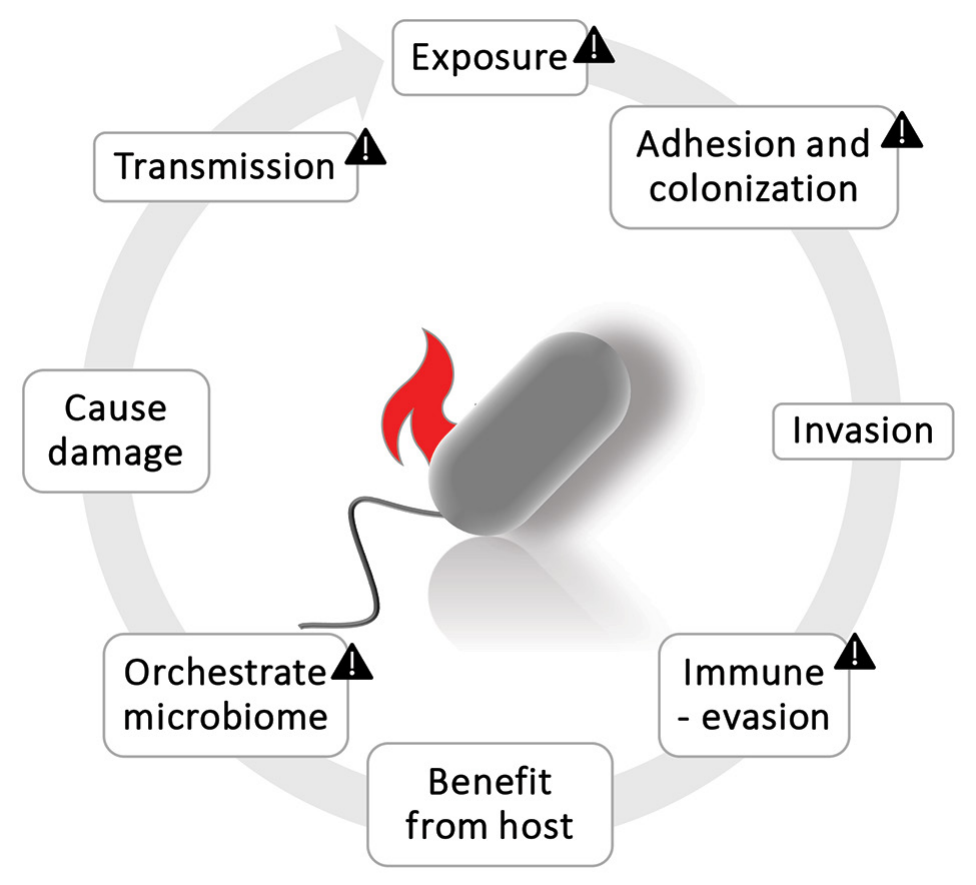
Cycle of pathogen actions. Triangles indicate pathogenic potentials for archaea. The other actions still require attention and strategic research. Figure created with BioRender (https://www.biorender.com).

Several steps are necessary for bacterial pathogenicity to develop: invasion, colonization, damage, and transmission (*16*) (Figure 1). Some of those traits overlap with archaeal characteristics, including exposure to the host (archaea are commensals) and the ability to adhere to and colonize the host (archaea form biofilms and might be able to attach to surfaces through their adhesins). Furthermore, the immunogenic reaction is highly species-specific; immunogenic potential is low for *Methanobrevibacter* spp. but stronger for *Methanosphaera* spp. (*17*). Transmission to other hosts seems probable; Pausan et al. demonstrated that methanogens can survive extended periods under oxygenated conditions (*18*). Archaea can team up with bacterial pathogens in harmful groups that can enter hosts’ tissues and benefit from invasion or cause damage to the host. In this context, we should mention the keystone pathogen hypothesis (*19*), in which some microorganisms could orchestrate the microbiome toward a disease status even in low numbers. Indeed, their metabolic capacity at the end of the food chain, consuming fermentation waste products, indicates that methanogens can influence the entire microbiome; their extraordinarily strong pull toward methane production can result in a microbial community optimized for fiber degradation and coping with B12 shortage (*6*).

Overall, the discussion on the potential of archaea for bearing pathogenic action at this stage remains highly speculative, underscoring the need for further research to elucidate their role in disease. We will address the different examples for infectious state in the human body that involve archaea and warrant deeper studies.

## Potential Contribution to Infectious Diseases

**Figure 2:**
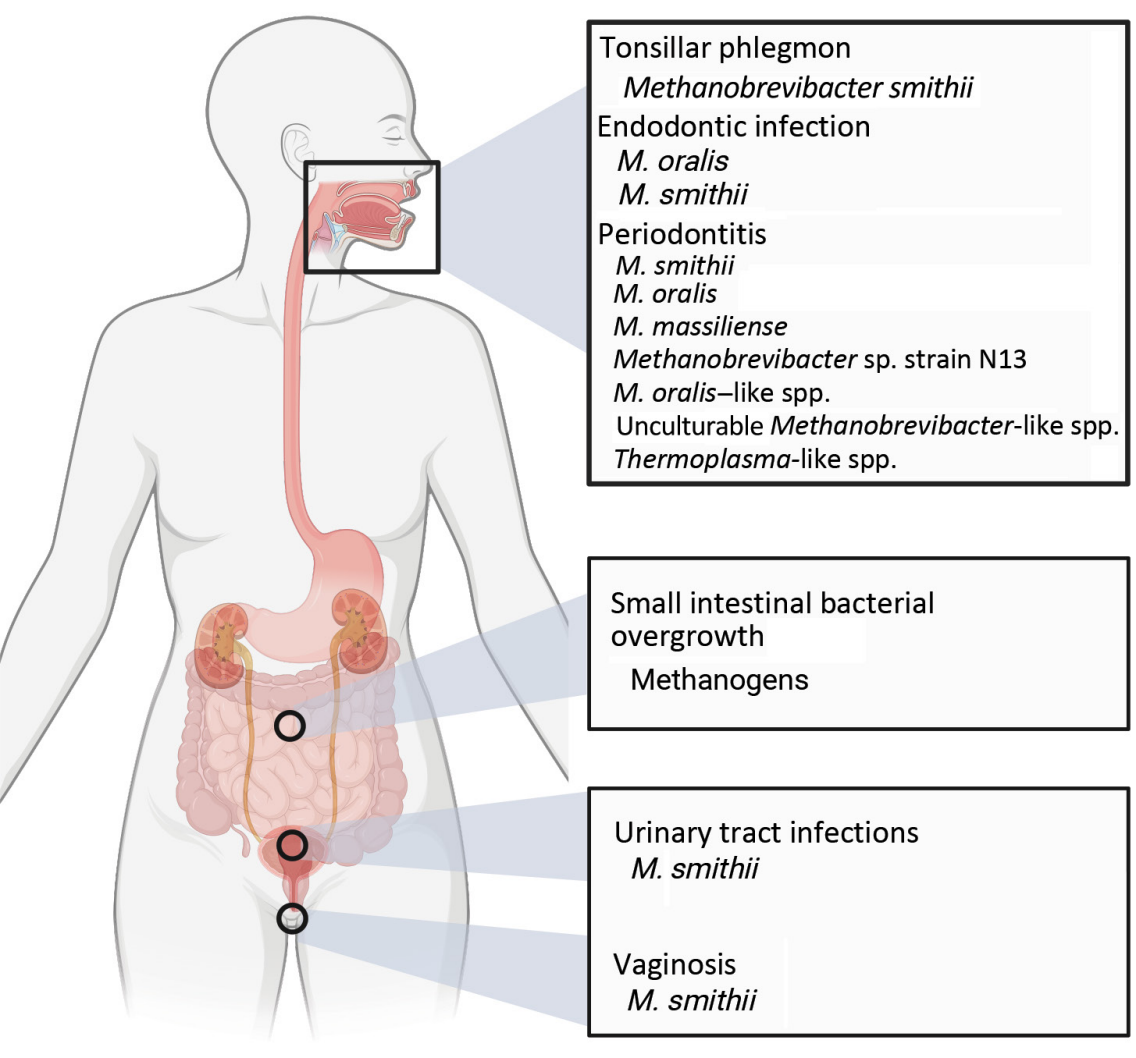
Infectious diseases in areas of the body in which archaea may have an effect. Figure created with BioRender (https://www.biorender.com).

The involvement of pathogenic bacteria in various infectious diseases is often the focus of studies, but our understanding of the role of archaea in this context is still limited. Although no pathogenic archaea are known to date, the literature has presented some evidence of links between archaea and various infectious diseases (*4*,*20*,*21*) (Figure 2).

Principles of archaeal syntrophy may play a role in shaping specific microbial networks that influence the overall condition but do not cause disease. Studies suggest that methanogenic archaea engage in a symbiotic relationship with bacteria; they use hydrogen (H_2_) and other byproducts of bacterial fermentation for methanogenesis, thereby reducing H_2_ pressure (*1*). In addition, they produce metabolites, including short-chain fatty acids or vitamins (e.g., formate, B12) (*6*) that could support the growth of both archaea and potentially pathogenic bacteria (*1*,*4*,*21*).

We will explore the potential role of archaea in human infectious diseases in the oral cavity, gastrointestinal tract, and urogenital tract. Archaea have also been detected in other parts of the body, such as the skin (*22*) and the respiratory tract (*3*). However, for both those sites, the information available is too sparse to elaborate on potential infectious traits.

### Oral Cavity

A diverse microbial community thrives in the oral cavity; it includes various microorganisms, among which archaea, specifically the genus *Methanobrevibacter*, should be emphasized. The archaea have been identified in both healthy and sick persons (*1*,*4*). Of note, the strain and species resolution of archaeal diversity associated with humans is only partially satisfactory so far. Most of the studies we cite used 16S rRNA gene sequencing (partially with short reads), which does not allow differentiation of the major *Methanobrevibacter* species. Therefore, any subsequent information provided at the species level should be carefully scrutinized and requires reevaluation.

#### Periodontitis

Periodontitis is one of the most common bacterial infections in humans and the 6th most common disease worldwide (*23*). It is a complex dysbiotic disease that develops over a longer timeframe and is the consequence of destructive host immune response to pathogenic, biofilm-forming microorganisms (*24*). It is characterized by the gradual loss of bone, the development of periodontal pockets, and the progressive decline in tooth function (*25*,*26*). Untreated periodontitis is recognized as a risk factor for life-threatening systemic diseases, such as endocarditis, atherosclerosis, and stroke. In addition, it is considered a co-factor for preterm birth (*25*).

Various microorganisms have been implicated in the onset of periodontitis, including different bacteria (e.g., *Tannerella*, *Treponema*, *Prevotella*, *Fusobacterium*), and potentially methanogenic archaea (*25*,*26*). In particular, *M. oralis*, *M. smithii*, and *M. massiliense* were identified in periodontitis patients within subgingival plaques and deep periodontal pockets (*27*). It has been shown that *M. oralis* is more prevalent and abundant in severe periodontitis but was not detected in healthy sites close-by and was not found after healing (*27*). Methanogenic archaea can comprise up to 18% of all community members in severe periodontitis (*1*,*28*,*29*).

Archaea have a completely different biology from bacteria. Most of the methods used in microbiology and microbiome research have been developed to optimally study and analyze bacteria, which means archaeal signatures are overlooked in most studies, so knowledge about the involvement of archaea is very limited (*8*). To date, the microbiology of periodontitis has been poorly studied and often remains descriptive. The reasons for the development of the various complex stages and disorders, and the influence of therapy and treatment, remain largely unexplored.

#### Endodontic Infection

Endodontic infection refers to a microbial infection that develops within the pulp of a tooth, the central part containing nerves, blood vessels, and connective tissues. Typically caused by bacterial invasion, such infections often stem from factors such as tooth decay, dental trauma, incorrect cavity preparation during dental procedures, or use of contaminated restorative materials. Bacteria can infiltrate the root canal primarily through caries, dental anomalies, lateral canals, or damaged cementum. The predominant bacterial phyla identified in infected root canals are Firmicutes, Proteobacteria, Spirochaetes, Bacteroidetes, and Actinobacteria (*30*).

Efenberger et al. detected methanogenic archaea, especially *M. smithii* and *M. oralis*, in 85% of infected pulp tissues using 16S rRNA gene sequencing. That finding suggests that archaea may play a role in the development of endodontic infection, potentially in collaboration with bacteria (*30*). Given that methanogens are involved in deep oral and dental infections, and considering the associated barrier breach, they could spread from that location to other closely located body areas and possibly lead to brain and muscular abscesses, as described in single cases (*31*). 

#### Tonsillar Phlegmon

Tonsillar phlegmon, a retropharyngeal abscess, mainly affects adolescents and young adults and is caused by an infection originating from the peritonsillar space and the pharyngeal sphincter. The infection can spread to adjacent tissues and, if not treated promptly, this condition, which often involves pathogens such as *Streptococcus pyogenes* and *Fusobacterium necrophorum*, can escalate and become a serious threat (*32*). 

Within tonsillar phlegmon, archaea, particularly *M. smithii*, may foster the growth of pathogenic bacteria through syntrophic interactions. In addition, archaeal methanogenesis activity, leading to methane (CH_4_) production, could contribute to radiologically visible gas in tonsillar phlegmon cases (*32*). Djemai et al. identified *M. smithii* using 16S rRNA gene sequencing with coexisting bacteria commonly found in *M. smithii* co-infections. Those bacteria belonged to enterobacterial and bacterial orders that include hydrogen producers. 

### Gastrointestinal Tract

The gastrointestinal tract houses most of the microbes in our body, including a rich diversity of archaea. Predominant members of the gastrointestinal archaeome include *M. smithii* and *Candidatus* M. intestini, constituting >90% of the gut archaeome (*4*,*5*). Additional noteworthy members encompass *M. stadtmanae*, *M. oralis*, Methanosarcinales, Methanomassiliicoccales, and Haloarchaea (*33*–*35*).

Methanogenic archaea are known for performing methanogenesis, converting bacterial end products (hydrogen and carbon dioxide) into methane and adenosine triphosphate (*1*,*36*). Methane travels through the gut and is expelled through flatus or enters the blood to eventually be excreted through the lungs; it is detectable in human breath (*37*). High methane breath levels (>5 ppm) were shown to correlate with a thousandfold increase in *Methanobrevibacter* abundance in the gut (*6*). Therefore, methane production serves as a reliable biomarker for the presence of methanogens in the gut. Varied breath methane levels (and corresponding methanogen abundances) have been associated with diverse health conditions, including small intestinal bacterial overgrowth (SIBO) (*4*).

#### SIBO

SIBO is characterized by an overgrowth of bacteria, surpassing normal levels (>10^5^ colony-forming units), in the small intestine (*38*). Usually, this overgrowth is caused by gram-negative anaerobic bacteria, manifesting by a hydrogen-positive breath test. In a substantial (30%) number of cases, the overgrowth of archaea was reported (as methane-dominant SIBO or intestinal methanogen overgrowth), recognized by the production of methane, instead of H_2_. Of note, SIBO is more prevalent in patients with inflammatory bowel syndrome than in healthy controls; a link with methanogen presence and constipation-type IBS has been established (*39*).

Madigan et al. suggested differing symptoms between SIBO caused by methanogens and that caused by hydrogen-producing bacteria (*40*). Methane-producing SIBO, especially prevalent in older persons, is linked to a reduced incidence of vitamin B12 (cobalamin) deficiency (*38*,*41*). Methanogens, which are able to produce cobalamin themselves (*42*), could help to make up for such a deficiency, which is in line with a previous study that showed that reduced vitamin B12 uptake was associated with a high methanogen load in the gastrointestinal tract (*6*,*40*). Conversely, hydrogenic SIBO is associated with higher occurrences of symptoms like diarrhea, cholecystectomy, diabetes, and Roux-en-Y gastric bypass surgery. Those distinctions may arise from varying sensitivities of archaeal physiology to host factors, including gut anatomy, motility, luminal bile acid concentration, and the capacity to synthesize or salvage cobalamin from neighboring microbiota or diet (*40*).

### Urogenital Tract

The urogenital tract encompasses a system of organs integral to urinary and reproductive functions. This system comprises the kidneys, ureters, bladder, and urethra to form, transport, and eliminate urine. Furthermore, it incorporates reproductive organs such as testes in male persons and the ovaries, uterus, and vagina in females (*43*). The urogenital microbiota contribute to the maintenance of homeostasis in the urinary tract by influencing the immune response; alterations of the microbial communities are documented to be related to various diseases (*44*). The close proximity of the urinary tract, vagina, and gastrointestinal tract enables the transmission of microorganisms, including archaea, from feces to those regions, which may contribute to infectious diseases such as urinary tract infections and vaginosis.

#### Urinary Tract Infections

Traditionally, urine was believed to be sterile; however, current understanding acknowledges the presence of a microbiome in the urogenital tract. This microbiome plays a crucial role in the pathophysiology of urinary tract infections (UTIs) and the maintenance of urinary tract health (*44**,**45*). UTIs commonly arise when microbes from the rectal area enter the urethra, migrating upward into the bladder or other areas of the urinary tract (*46*).

Various factors, including sexual activity, urinary tract abnormalities, a weakened immune system, catheter use, and hormonal changes, can elevate the risk of a UTI developing (*47*). Bacteria are typically associated with UTIs; however, *M. smithii* has also been identified as a component of the urinary microbial community. Urine samples containing *M. smithii* have exhibited the presence of hydrogen-producing enterobacteria, including *Escherichia coli* and *Klebsiella pneumoniae*. The consistent co-occurrence suggests a potential role of *M. smithii* in supporting the dysbiosis by promoting the growth of enterobacteria, which are recognized agents of UTIs (*45**,**46*).

#### Vaginosis

Vaginosis refers to an imbalance in the vaginal microbiota, leading to discomfort and noticeable symptoms (*48*). The vagina typically hosts microbiota with lower diversity, predominantly consisting of lactobacilli. Vaginosis can arise as a result of changes in vaginal pH or transfer of fecal microbes from the gastrointestinal tract to the vagina (*48*). Of note, *M. smithii* has been identified in patients with vaginosis, and its presence has been proposed as a potential biomarker for this condition (*48*). This association is plausible, considering that *M. smithii* is the most prevalent archaea found in the gastrointestinal tract (*4*). Some studies have faced challenges in detecting archaea in samples from both healthy and infected women (*49*). It has been suggested that methanogens contribute to vaginal health (C.J. Neumann et al., unpub. data, https://www.biorxiv.org/content/10.1101/2023.08.31.555744v1).

Methanogenesis by archaea is believed to play a role in maintaining the pH balance of the vaginal microbiome. Moreover, it helps prevent the accumulation of acidic conditions that could disrupt the vaginal microbiome (*50*). Although *M. smithii* might be among the usual vaginal microbiota, its specific connection to vaginosis requires further investigations for a comprehensive understanding.

## Conclusions

The literature demonstrates that archaea constitute active components within the human microbiome and are prevalent across a range of different body sites (*2*). Associations with various infectious diseases, such as periodontitis and endodontic infections within the oral cavity, SIBO in the gastrointestinal tract, and infections of the urogenital tract, have been reported (*1*,*4*). Although many of those reports are preliminary and do not fully resolve the archaeal components taxonomically, the genus *Methanobrevibacter* emerges as the most commonly observed archaea possibly linked to these infectious diseases (Figure 2; Appendix).

Understanding the complexities of detoxifying microbial metabolic products, particularly in the context of methanogenesis, carries substantial implications for both pathogen growth and disease onset. The interaction between archaea and bacteria in syntrophic relationships offers valuable insights into the dynamics within microbial communities. It is essential to conduct a thorough investigation to reveal the complex mechanisms behind these connections, which could play a role in shifting the microbiome toward a diseased state. Those experiments not only would shed light on potential disease mechanisms but also could open up paths for novel therapeutic approaches to address microbial dysbiosis linked to conditions such as periodontitis and other diseases (*50*).

The extent to which interactions with archaea are beneficial, neutral, or pathogenic is not well understood. Furthermore, advancing research in human health demands an increased focus on clinical studies that consider the inflammatory potential of archaea. In addition, such studies should explore the influence of archaeal metabolisms and their products, including the effects of methane on the human body (*1*). Although archaea are generally considered nonpathogenic, they could have the potential prerequisites for pathogenesis. Various hypotheses, such as the not-yet-been-discovered, metabolism, gene-absence, and virus hypotheses, are proposed to explain the apparent absence of archaeal pathogens (*8*,*10*,*11*,*16*). However, because of limited knowledge, challenges in gene annotation, and the complex nature of archaeal behavior in host environments, those hypotheses have not been proven. Although some pathogenic traits overlap between bacteria and archaea, the discussion of potential pathogenic action of archaea is ongoing and emphasizes the need for further research to understand their role in disease.

To gain a more comprehensive understanding of the role of archaea in health and disease requires additional initiatives aimed at effectively characterizing host-associated and disease-associated archaea. Despite the use of molecular biologic techniques in studying the archaeome, detecting archaea presents challenges primarily because standard protocols in research and diagnostics that are optimized for bacteria prove suboptimal for archaea because of their diverse cell structure, physiology, and metabolic activity. Those distinctive features, coupled with inappropriate protocols for sampling, DNA extraction, and PCR primer selection, create obstacles in visualizing, culturing, or analyzing archaea. Moreover, the absence of extensive reference databases hinders a thorough evaluation of the acquired data. Compounding this challenge is the presence of a robust bacterial or host background, further complicating the detection and analysis of archaea (*8*). 

**Figure 3:**
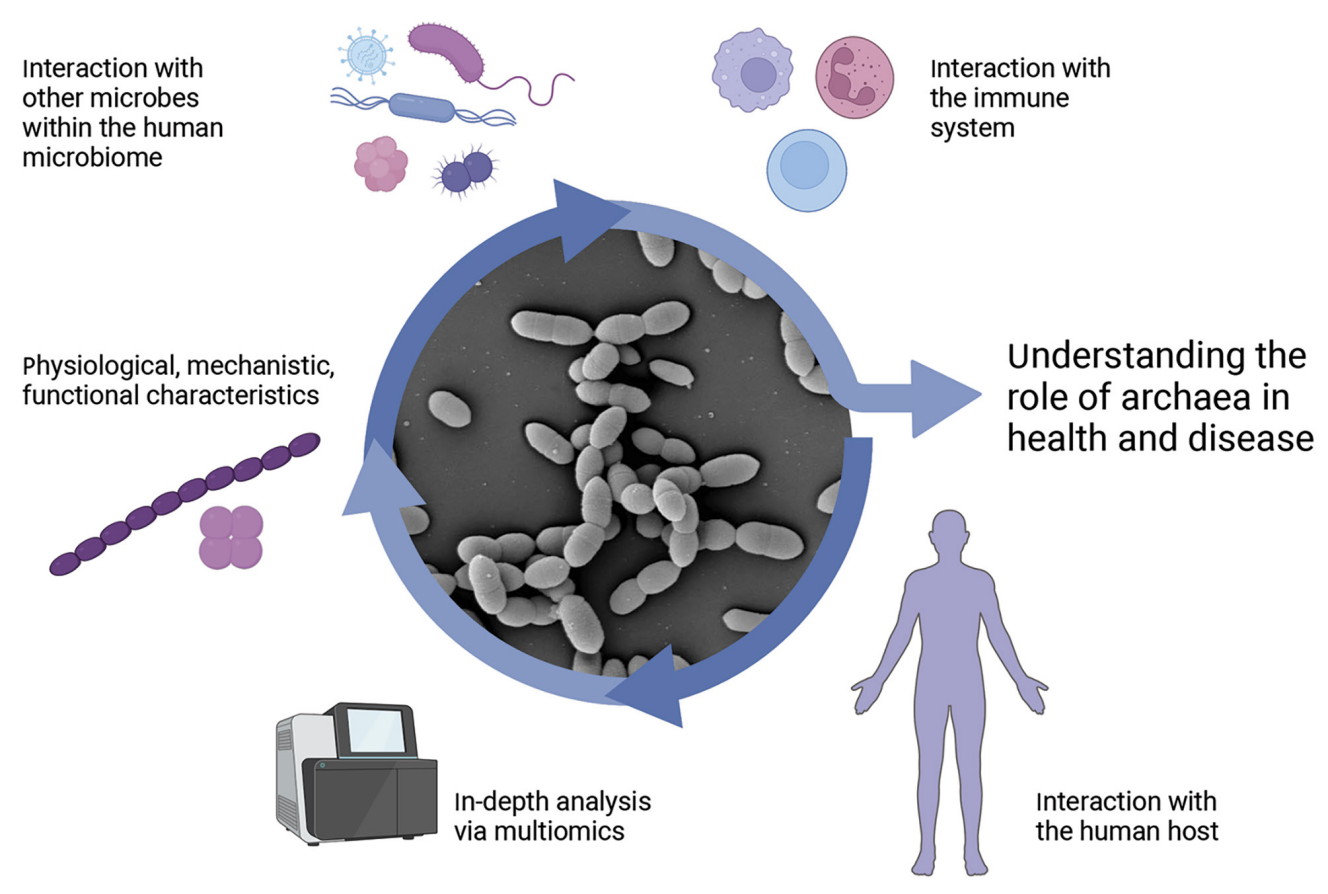
Exploration of analytical potentials using archaeal isolates to enhance understanding of the role of archaea in health and disease. Figure created with BioRender (https://www.biorender.com).

The limited availability of archaeal cultures hinders in-depth, mechanistic, physiologic and comprehensive analyses, including multiomics and interaction studies that could deepen our comprehension of archaea’s role in health and disease (Figure 3). According to the Global Catalog of Microorganisms (https://gcm.wdcm.org/species?taxonid=2173), only a handful of host-associated archaeal isolates are publicly accessible in culture. Those isolates include 3 strains of *M. oralis* from the human oral cavity and subgingival plaque, along with 4 strains of *M. smithii* isolated from human feces and the large intestine. The limited availability of cultures presents a substantial hindrance to conducting thorough research. Publications do exist describing the successful isolation of various archaea from patients with diverse health conditions (*5*), but there is currently no comprehensive culture collection for archaea. Establishing such a collection would be pivotal for advancing research on the archaeome; we have established a cultivation pipeline for the enrichment and cultivation of methanogenic archaea in a highly efficient way (J. Duller, et al., unpub. data, https://doi.org/10.1101/2024.04.10.588852).

Expanding our collection of human-derived isolates is crucial for gaining deeper insights into the functions and roles of archaea, enabling functional analyses in disease models, and determining the effects of their structural elements and metabolites. Microorganisms within a microbiome network engage in various interactions that affect each other and the health of the host. Understanding of such microbial interactions beyond the bacterial sphere is essential for a more comprehensive grasp of disease mechanisms and the search for new diagnostic, preventive, and therapeutic strategies.

Appendix Additional information about potential effects of archaea on human infectious disease.
