# Supplementary material for: Archaea in the Human Microbiome and Potential Effects on Human Infectious Disease
Source: Emerg Infect Dis. 2024 Aug;30(8):1505–13. doi: 10.3201/eid3008.240181 (PMC11286065; doi:10.3201/eid3008.240181)
Supplement: Appendix — Additional information about potential effects of archaea on human infectious disease. [file 24-0181-Techapp-s1.pdf]

Article DOI: http://doi.org/10.3201/eid3008.240181

EID cannot ensure accessibility for supplementary materials supplied by authors. Readers who have difficulty accessing supplementary content should contact the authors for assistance.

Archaea in the Human Microbiome and Potential Effects on Human Infectious Disease

Appendix

Additional Information on *Methanobrevibacter* Species

Methanobrevibacter species demonstrate remarkable adaptability to engage with both hosts and the non-archaeal elements within their microbiomes. These members of the Methanobacteriaceae family (Methanobacteriota phylum), are characterized by their ability to perform methanogenesis from bacterial fermentation products, namely H2 and CO2. Formate and methanol could be further substrates, as their utilization is genetically encoded (*1*). In addition, *Methanobrevibacter* spp. exhibit various adaptations to the human gut, including the formation of adhesins (*2*), and the presence of genes encoding bile salt hydrolases (*3*). *Methanobrevibacter* species dominate the archaeal population in the gastrointestinal tracts (GITs) of various ruminant and non-ruminant animals, and are even associated with plants and their microbiomes (summarized in (*4*)). Although *Methanobrevibacter* species are very abundant in ruminants, to our knowledge they have not been reported to cause inflammation or disease in these animals, limiting interest primarily to the reduction of methane emissions (*5*). Not only in this aspect, *Methanobrevibacter* bacterial interactions are of high interest, which is why specific cocultures of bacteria (e.g., *Christensenella minuta*, *Bacteroides thetaiotaomicron*) (*6–8*) and *M. smithii* have been established, indicating a fine-tuned collaboration with effects on each other's metabolism. For instance, *C. minuta* shifted its metabolisms rather toward acetate than butyrate in the presence of *M. smithii*, which uses acetate for biomass production (*7*).

References

1. Chibani CM, Mahnert A, Borrel G, Almeida A, Werner A, Brugère JF, et al. A catalogue of 1,167 genomes from the human gut archaeome. Nat Microbiol. 2022;7:48–61. PubMed https://doi.org/10.1038/s41564-021-01020-9

2. Samuel BS, Hansen EE, Manchester JK, Coutinho PM, Henrissat B, Fulton R, et al. Genomic and metabolic adaptations of *Methanobrevibacter smithii* to the human gut. Proc Natl Acad Sci U S A. 2007;104:10643–8. PubMed https://doi.org/10.1073/pnas.0704189104

3. Jones BV, Begley M, Hill C, Gahan CGM, Marchesi JR. Functional and comparative metagenomic analysis of bile salt hydrolase activity in the human gut microbiome. Proc Natl Acad Sci U S A. 2008;105:13580–5. PubMed https://doi.org/10.1073/pnas.0804437105

4. Borrel G, Brugère JF, Gribaldo S, Schmitz RA, Moissl-Eichinger C. The host-associated archaeome. Nat Rev Microbiol. 2020;18:622–36.

5. Tseten T, Sanjorjo RA, Kwon M, Kim SW. Strategies to mitigate enteric methane emissions from ruminant animals. J Microbiol Biotechnol. 2022;32:269–77. PubMed https://doi.org/10.4014/jmb.2202.02019

6. Catlett JL, Carr S, Cashman M, Smith MD, Walter M, Sakkaff Z, et al. Metabolic synergy between human symbionts *Bacteroides* and *Methanobrevibacter.* Microbiol Spectr. 2022;10:e0106722. PubMed https://doi.org/10.1128/spectrum.01067-22

7. Ruaud A, Esquivel-Elizondo S, de la Cuesta-Zuluaga J, Waters JL, Angenent LT, Youngblut ND, et al. Syntrophy via interspecies H_2 transfer between *Christensenella* and *Methanobrevibacter* underlies their global cooccurrence in the human gut. MBio. 2020;11:e03235-19. PubMed https://doi.org/10.1128/mBio.03235-19

8. Samuel BS, Gordon JI. A humanized gnotobiotic mouse model of host-archaeal-bacterial mutualism. Proc Natl Acad Sci U S A. 2006;103:10011–6. PubMed https://doi.org/10.1073/pnas.0602187103
